# Special Issue Editor’s choice: Global child health from birth to adolescence and beyond

**DOI:** 10.1371/journal.pmed.1003800

**Published:** 2021-09-28

**Authors:** Caitlin Moyer

**Affiliations:** Public Library of Science, San Francisco, California, United States of America and Cambridge, United Kingdom

## Abstract

Caitlin Moyer discusses PLOS Medicine’s Special Issue on Global Child and Adolescent Health.

In a new PLOS Medicine special issue, entitled “Global Child and Adolescent Health: From Birth to Adolescence and Beyond”, guest editors Zulfiqar Bhutta, Quique Bassat, and Kathryn Yount bring to the forefront new research to illuminate global challenges for achievement of child and adolescent health and survival goals, and novel ways to address these challenges. The research articles in this special issue cover a broad range of current threats to child and adolescent health and wellbeing around the globe, shining a spotlight on the health impacts of pollutions, climate change, injury, violence, infectious diseases, undernutrition, and adolescent pregnancy. It is important to identify the gaps in knowledge that stand in the way of ensuring that all children and adolescents have the opportunity to survive, grow, and achieve developmental, social, and academic milestones. These studies further this goal by investigating new strategies that may be adopted to address these areas of need.

## Infant mortality

Preterm birth contributes a substantial burden of child mortality around the globe, and strategies to improve the health and survival of children born preterm are imperative. In a community-based, cluster randomized trial, Aarti Kumar and colleagues investigated the effectiveness of a topical sunflower seed oil therapy combined with gentle massage in reducing neonatal mortality in Uttar Pradesh, India [1]. The study was set in the community, and the intervention was delivered by families and compared with usual newborn skin care practices, making it distinct from previous hospital-based investigations. Although the intervention did not lead to significant differences in neonatal mortality at 24 hours after birth, the findings from additional analyses suggested improvements in mortality among very low birthweight infants, who are often born preterm. Thus, further study is warranted to investigate sunflower seed oil therapy as a strategy to reduce the mortality rate among low birth weight or preterm infants.

Several articles in this issue touch on the burden and associated outcomes of infections during early childhood. A substantial proportion of deaths among children under 5 years of age are related to preventable or treatable infections, including pneumonia, sepsis, diarrhea, and malaria. Malaria is a significant driver of child mortality across the globe, and in malaria endemic regions, women are at risk of infection during pregnancy. In a randomized trial, D. Taylor Hendrixson and colleagues tested an intervention combining ready-to-use supplementary food, azithromycin, and intermittent testing and treatment for malaria and vaginal dysbiosis on birth outcomes in Sierra Leone [2]. The study revealed that infants born to undernourished pregnant women receiving the intervention had an increase in birth weight and length, and fewer infants died. The findings suggest that interventions combining supplementary food and measures to control common infections may be beneficial as measures to prevent neonatal mortality.

## Undernutrition and child growth

Undernutrition is a significant child health risk, associated with 45% of child deaths according to the World Health Organization, and includes wasting, micronutrient deficiency, and sub-optimal breastfeeding [3]. It is important to demonstrate how strategies to improve child nutrition can be implemented in challenging contexts, with limited resources. In a trial conducted in South Africa, Maya Adam and colleagues investigated the effectiveness of an animated video intervention, delivered by community health workers to mothers in home settings to promote breastfeeding practices and knowledge in South Africa [4]. The results suggested that the impact of the intervention on exclusive breastfeeding and maternal knowledge was similar to that in mothers who received standard of care engagement with the “mentor mother” community health workers. This is important, as it suggests the potential for mobile video- based intervention approaches to be a useful surrogate for face-to-face interaction with community health workers in settings where community health works may not be available.

One study featured in this issue describes a multi-faceted intervention to address the challenge of improving child growth in regions where infections are common, often going hand-in-hand with prevalent child stunting. In a randomized trial, Mark DeBoer and colleagues investigated the effect of antimicrobial and nicotinamide interventions to promote growth of children from birth through 18 months of age in Tanzania [5]. While neither scheduled administration of azithromycin and nitazoxanide, or daily nicotinamide supplementation was shown to be effective in improving child growth, the findings of the study provide valuable insight into the gaps that remain to be addressed in identifying new approaches to address interrelated issues of enteric infection, child growth, and nutrient deficiency in similar settings. Taken together, several of the articles shed new light on the potential to address child mortality and developmental outcomes by reducing the risks of undernutrition and infection early in life.

## Adolescent violence and mental health

Adolescence represents a particularly dynamic stage of human development; however, adolescents are underrepresented in many research contexts, and often fewer health-related services are available for this age group. Papers in this issue delve into some of the current challenges to adolescent health, including serious injury, HIV infection, and experiences of violence. In an analysis of the Creating Opportunities through Mentorship, Parental Involvement, and Safe Spaces (COMPASS) program intervention trial findings, Ilana Seff and colleagues studied the associations between changes in gender attitudes among caregivers and violence victimization and schooling of adolescent girls in South Kivu, Democratic Republic of Congo [6]. The intervention included a caregiver curriculum on top of a 12-month life skills program delivered to adolescent girls. Over the course of the intervention, changes in caregiver attitudes were evaluated, and increases in gender-equitable attitudes among caregivers was associated with greater likelihood of school participation. Changes in caregiver attitudes were also associated with greater odds of girls experiencing a decline in physical violence over the course of the study, though this association did not reach statistical significance. Results of this study support the notion that strategies that improve caregiver gender-equitable attitudes could be a route to helping adolescent girls thrive academically, and may also reduce the exposure of adolescent girls to physical violence.

New strategies are necessary to tackle the rise of mental health issues around the globe, including among children and adolescents [7]. There is a need to promote interventions for adolescent mental health that are effective both in the short term and are additionally able to drive sustained effects in the longer term. To address this goal, in a 12 month trial extension, Kanika Malik, Daniel Michelson, and colleagues investigated the sustained effectiveness of a lay counsellor-delivered problem solving skill-based intervention to promote mental health of adolescents enrolled in low-income schools in New Delhi, India [8]. When participants were followed up after 12 months, those among the intervention group exhibited small but sustained improvements in self-reported psychosocial problems and mental health symptoms, and an economic analysis was supportive of the potential to scale up such an intervention, potentially supporting school-based delivery of interventions to promote availability of mental health services to adolescents in similar settings. The findings of this follow up provide welcome insight into how brief, low-cost mental health interventions may translate into sustained improvement of adolescent mental health needs in low-resource contexts.

Together, these studies provide an encouraging snapshot of new strategies targeted toward improving child survival, growth, and well-being across diverse contexts globally. The findings from these and other papers included in this special issue should encourage research across diverse global settings, and inspire the research community to explore new relationships and factors that will build the foundation for future interventions to target child and adolescent health and support universal achievement of developmental, social, and academic milestones. The interventions and strategies currently being investigated will serve to advance child health across a variety of challenging contexts going forward. We look forward to reporting on future advances in research exploring novel strategies to ensure that children and adolescents across the globe have the opportunity to grow and thrive.

## References

[1] Kumar A, Mishra S, Singh S, Ashraf S, Kan P, Ghosh AK, et al. Effect of sunflower seed oil emollient therapy on newborn infant survival in Uttar Pradesh, India: A community-based, cluster randomized, open-label controlled trial. PLoS Med. 2021;18(9): e1003680. 10.1371/journal.pmed.1003680 PMC8478176 34582448

[2] Hendrixson DT, Smith K, Lasowski P, Callaghan-Gillespie M, Weber J, Papathakis P, et al. A novel intervention combining supplementary food and infection-control measures to improve birth outcomes in undernourished pregnant women in Sierra Leone: A randomized, controlled clinical effectiveness trial. PLoS Med. 2021;18(9): e1003618. 10.1371/journal.pmed.1003618 PMC8478228 34582451

[3] World Health Organization. Malnutrition. 2021 Jun 9 [cited 7 September 2021]. Available from: https://www.who.int/news-room/fact-sheets/detail/malnutrition

[4] Adam M, Johnston J, Job N, Dronavalli M, Le Roux I, Mbewu N, et al. Evaluation of a community-based mobile video breastfeeding intervention in Khayelitsha, South Africa: The Philani MOVIE cluster-randomized controlled trial. PLoS Med. 2021;18(9): e1003744. 10.1371/journal.pmed.1003744 PMC8478218 34582438

[5] DeBoer MD, Platts-Mills JA, Elwood SE, Scharf RJ, McDermid JM, Waniuhi AW, et al. Effect of scheduled antimicrobial and nicotinamide treatment on linear growth in children in rural Tanzania: A factorial randomized, double-blind, placebo-controlled trial. PLoS Med. 2021;18(9): e1003617. 10.1371/journal.pmed.1003617 PMC8478246 34582462

[6] Seff I, Falb K, Yu G, Landis D, Stark L. Gender-equitable caregiver attitudes and education and safety of adolescent girls in South Kivu, DRC: A secondary analysis from a randomized controlled trial. PLoS Med. 2021;18(9): e1003619. 10.1371/journal.pmed.1003619 PMC8478225 34582454

[7] Patel V, Saxena S, Lund C, Thornicroft G, Baingana F, Bolton P, et al. The Lancet Commission on global mental health and sustainable development. Lancet. 2018; Oct 27;392(10157):1553–1598. doi: 10.1016/S0140-6736(18)31612-X Epub 2018 Oct 9. Erratum in: Lancet. 2018 Oct 27;392(10157):1518. . 30314863

[8] Malik K, Michelson D, Doyle AM, Weiss HA, Greco G, Sahu R, et al. Effectiveness and costs associated with a lay counsellor delivered, brief problem-solving mental health intervention for adolescents in urban, low-income schools in India: 12-month outcomes of a randomized controlled trial. PloS Med. 2021;18(9): e1003778. doi: 10.1371/journal.pmed.1003778 PMC8478208 34582460

